# Phenotype, functions and fate of adoptively transferred tumor draining lymphocytes activated ex vivo in mice with an aggressive weakly immunogenic mammary carcinoma

**DOI:** 10.1186/1471-2172-11-54

**Published:** 2010-11-04

**Authors:** Catriona HT Miller, Laura Graham, Harry D Bear

**Affiliations:** 1 Department of Microbiology and Immunology, Virginia Commonwealth University's Medical College of Virginia, Richmond, Virginia, USA; 2 Division of Surgical Oncology, Department of Surgery, Virginia Commonwealth University's Medical College of Virginia and the Massey Cancer Center, Richmond Virginia, USA

## Abstract

**Background:**

Regression of established tumors can be induced by adoptive immunotherapy with tumor draining lymph node lymphocytes activated with bryostatin and ionomycin. We hypothesized that tumor regression is mediated by a subset of the transferred T lymphocytes, which selectively infiltrate the tumor draining lymph nodes and proliferate *in vivo*.

**Results:**

Adoptive transfer of B/I activated tumor draining lymphocytes induces regression of advanced 4T1 tumors, and depletion of CD8, but not CD4 T cells, abrogated tumor regression in mice. The predominant mediators of tumor regression are CD8+ and derived from CD62L^- ^T cells. Transferred lymphocytes reached their peak concentration (10.5%) in the spleen 3 days after adoptive transfer and then rapidly declined. Adoptively transferred cells preferentially migrated to and/or proliferated in the tumor draining lymph nodes, peaking at day 5 (10.3%) and remained up to day 28. CFSE-stained cells were seen in tumors, also peaking at day 5 (2.1%). Bryostatin and ionomycin-activated cells proliferated vigorously *in vivo*, with 10 generations evident in the tumor draining lymph nodes on day 3. CFSE-stained cells found in the tumor draining lymph nodes on day 3 were 30% CD8^+^, 72% CD4^+^, 95% CD44^+^, and 39% CD69^+^. Pre-treatment of recipient mice with cyclophosphamide dramatically increased the number of interferon-gamma producing cells.

**Conclusions:**

Adoptively transferred CD8+ CD62L^low ^T cells are the principal mediators of tumor regression, and host T cells are not required. These cells infiltrate 4T1 tumors, track preferentially to tumor draining lymph nodes, have an activated phenotype, and proliferate *in vivo*. Cyclophosphamide pre-treatment augments the anti-tumor effect by increasing the proliferation of interferon-gamma producing cells in the adoptive host.

## Background

Conventional therapies for cancer, including surgery, radiation and chemotherapeutic agents, are often ineffective at controlling the growth and spread of tumors. The immune system can potentially eliminate cancerous cells, as demonstrated by studies of numerous animal models and a few clinical trials [1,2]. In most cases, it is thought that anti-tumor effects are mediated by cytotoxic T lymphocytes (CTL), which recognize MHC class I-peptide complexes on the tumor cell surface [3]. Monoclonal antibodies, cytokines, and pharmacological methods have been used successfully in mouse models to activate lymphocytes isolated from tumors or tumor draining lymph nodes (DLN), which can then be adoptively transferred to tumor bearing hosts and cause regression of established tumors [4-15]. Adoptive immunotherapy (AIT), or the adoptive transfer of antigen-sensitized T cells activated and/or expanded *in vitro *continues to receive attention [10][16-23].

We have shown that *in vitro *treatment with bryostatin and ionomycin (B/I) selectively activates antigen-sensitized tumor draining lymph node (tDLN) lymphocytes [19-22]. Bryostatin 1 is a macrocyclic lactone derived from Bugula neritina, a marine invertebrate. Bryostatin activates protein kinase C [23-26] and ionomycin increases intracellular calcium [27]. Together, these mimic signaling through the CD3/TcR complex and lead to activation and proliferation of T cells [24,27].

Previous research in our lab has shown that adoptive transfer of B/I activated tumor draining lymphocytes can cure subcutaneous tumors and visceral metastases in murine hosts and establish long-term immunity, without evidence of autoimmunity. In the 4T1 mammary carcinoma model, we have shown that B/I selectively activates CD62L^- ^or sensitized T cells and that the anti-tumor activity resides in the CD62L^- ^fraction of lymphocytes obtained from donor lymph nodes; only the CD62L^- ^subset proliferates after B/I activation and has anti-tumor activity [28]. CD62L or L-selectin is an adhesion molecule important in T cell homing to lymph nodes and is down-regulated after T cells are activated and differentiate into their effector or effector memory (T_EM_) phenotypes [29,30]. Thus, because of this selective activation of antigen-sensitized T cells from the vaccinated donor mice, B/I stimulated DLN lymphocytes have tumor antigen specific activity *in vivo*, despite the non-specific stimulus used to promote their growth.

We hypothesized that B/I activated T cells mediate tumor regression primarily by CD8^+ ^T cell mediated functions and may establish T cell memory in the adoptive host by proliferating *in vivo*. B/I activated cells were characterized prior to adoptive transfer, and the most active subsets of cells identified by depletion or separation of phenotypically distinct subsets of T cells prior to AIT. By AIT using CFSE-labeled cells, we were also able to determine the trafficking patterns and measure the *in vivo *proliferation of B/I-activated T cells in tumor-bearing hosts.

## Methods

### Mice

Virus-free BALB/c mice (Charles River Laboratories, Cambridge, MA) were used between 8 and 12 weeks of age, caged in groups of 6 or fewer, and provided food and water *ad libitum. *Nude athymic BALB/c mice (National Cancer Institute, Bethesda, MD) were used to produce hybridoma ascites. All guidelines of the Virginia Commonwealth University Institutional Animal Care and Use Committee, which conform to the American Association for Accreditation of Laboratory Animal Care and the U.S. Department of Agriculture recommendations for the care and humane experimental use of animals, were followed.

### Tumor cell lines and Hybridomas

4T1 mammary tumor cell line was kindly provided by Dr. Jane Tsai at the Michigan Cancer Foundation, Detroit, Michigan. Cells were maintained in Dulbecco's Modified Essential Medium (DMEM) with 10% heat-inactivated fetal calf serum (Hyclone, Logan, UT), 1 mM sodium pyruvate, 100 U/ml penicillin and 100 μg/ml streptomycin (Sigma, St. Louis MO) (modified DMEM). Meth A sarcoma, an unrelated tumor cell line (ATCC, Rockville, MD) was maintained in RPMI 1640 medium with 10% heat-inactivated FCS, 1 mM sodium pyruvate, 0.1 mM nonessential amino acids, 2 mM L-glutamine, 100 U/ml penicillin, 100 μg/ml streptomycin, 10 mM Hepes buffer, and 5 × 10^-5 ^M 2-mercaptoethanol (Sigma). Tumor cells were harvested for inoculation of mice with 0.05% trypsin-EDTA (Fisher, Pittsburgh, PA). Hybridomas (GK1.5 (anti-CD4), 2.43 (anti-CD8)) were obtained from ATCC and grown in complete RPMI. All cells were incubated in 250 ml T-flasks (PGC, Gaithersburg, MD) at 37°C in humidified air with 5% CO_2_.

### Monoclonal Antibody production

Anti-CD4 monoclonal antibody (mAb) and anti-CD8 mAb were produced as ascites fluid from pristane-primed nude mice injected with their respective hybridomas.

### Draining lymph node sensitization

Donor mice were vaccinated in the left hind footpad with 1 × 10^6 ^4T1 cells. Ten days after footpad vaccination, popliteal tumor draining lymph nodes (tDLN) were harvested under sterile conditions.

### Lymphocyte activation and *in vitro *expansion

DLN's were harvested and dispersed into a single cell suspension in complete RPMI media at 1 × 10^6 ^cells/ml. The cells were activated by incubation with 5 nM bryostatin 1 (provided by the National Cancer Institute, Bethesda, MD) and 10 nM ionomycin (Calbiochem, San Diego, CA) (B/I), and 80 U/ml of rIL-2 (Chiron, Emeryville, CA) at 37°C for 18 hours. Cells were washed three times with warm complete RPMI and resuspended at 1-2 × 10^6 ^cells/ml with 40 U/ml of rIL-2. The cells were allowed to proliferate in culture for an additional 7 days and were split every 2-3 days in order to maintain 1-2 × 10^6 ^cells/ml concentration.

### Adoptive immunotherapy

Host mice were inoculated in the left flank with 2.5 × 10^4 ^- 5 × 10^4 ^4T1 cells (2.5 × 10^4 ^for 7-10 day tumors, 5 × 10^4 ^for 4 day tumors). One day prior to AIT, mice were pretreated with cyclophosphamide (CYP),100 mg/kg IP(Mead Johnson, Princeton, NJ). On day 4, 7 or 10, the B/I activated and expanded DLN lymphocytes were washed twice in serum free medium (RPMI 1640) and injected intravenously (IV) in 0.5 ml into host mice. No systemic cytokines were administered.

### CFSE staining and analysis

Prior to adoptive immunotherapy, B/I activated DLN lymphocytes were stained with 50 μM CFDA-SE (Molecular Probes, Eugene, OR) in PBS at a concentration of 75 million cells/ml for 15 minutes. Cells were washed with warm media and incubated at 37°C for 30 minutes to allow processing by intracellular proteases. CFSE-stained lymphocytes were injected IV into CYP-treated or untreated mice bearing 4T1 tumors.

### Flow cytometry

Cells isolated from spleen, tumor, inguinal tDLN, and cLN of control or treated mice at various time points were stained with a panel of antibodies and analyzed by dual color flow cytometry for CFSE and surface marker expression on an ELITE Beckman Coulter flow cytometer. Fluorescently labeled Abs directed against the following markers were obtained from Pharmingen (San Diego, CA): Pan-DX5(DX5), CD4 (GK1.5), CD8 (53-6.7), CD44 (IM7), CD62L (MEL-14), and CD69 (H1.2F3). Appropriate isotype controls were used in all cases. Generations of proliferation detected by CFSE fluorescence were analyzed using ModFit LT (Verity Software House, Topsham, Maine) and acceptable fits were determined by reduced Chi-Squared values.

### *In vitro *T cell subset depletion experiments

For *in vitro *depletion studies, DLN were first incubated with antibody (1:100) for 30 minutes, washed, and then incubated with rabbit complement (C') (Accurate Chemical) at 37°C for 30 minutes. The efficacy of the depletion was tested by flow cytometry. To determine the CD62L phenotype of the sensitized T cell precursors that were activated by B/I to become anti-tumor effectors or of the cells after B/I activation that mediated anti-tumor effects in adoptive hosts, DLN cells before and/or after activation with B/I were separated into CD62L^- ^and CD62L^+ ^subsets using magnetic bead separation (EasySep, Stem Cell Technologies). Flow cytometry was used to verify fractionation.

### Tumor measurements

In all AIT experiments, tumor growth was monitored with biweekly measurements of perpendicular diameters. Results are reported as the mean tumor area ± standard error (SE). When the tumor area was greater than 100 mm^2 ^or if a mouse appeared ill, the animal was euthanized by CO_2 _inhalation. Complete tumor regression was defined as the absence of a measurable tumor on three consecutive measurements.

### Cytokine release assays

Interferon-γ (IFN-γ) release from tumor sensitized, fresh or B/I activated and expanded lymphocytes in response to stimulation with irradiated 4T1 and irradiated Meth A for 24 hours was assayed using ELIspot assays from Pharmingen (San Diego, CA).

### Statistical analysis

Differences in tumor growth were assessed by analysis of variance (ANOVA) and Tukey-Kramer honestly significant difference test (Tukey's HSD) using JMP_IN _software (SAS Institute Inc., Cary, N.C.). *In vivo *experiments included at least six mice per group and were repeated at least twice. A p < 0.05 was used throughout to determine significant differences.

## Results

### Adoptive transfer of tDLN activated by B/I and expanded *in vitro *induces regression of 10 day 4T1 tumors

**Figure 1:**
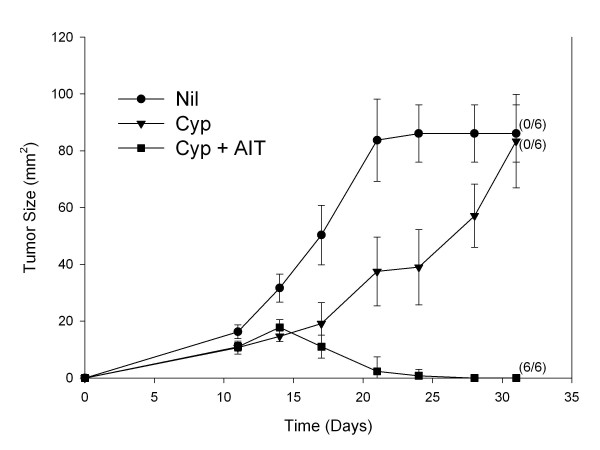
**Adoptive transfer of bryostatin and ionomycin activated tumor DLN lymphocytes induces regression of 10 day 4T1 tumors**. Mice with established 9 day tumors either were untreated or were treated with CYP (100 mg/kg i.p. on day 9), alone, or CYP followed by adoptive transfer of 10 × 10^6 ^4T1 DLN lymphocytes (on day 10) which had been activated with B/I and expanded in culture. Mean tumor areas ± SE are charted over time versus days after tumor inoculation. Treatment with CYP + B/I activated lymphocytes induced complete tumor regressions in 100% of the mice. CYP treatment alone slowed tumor growth only temporarily. Numbers to the right indicate the number of mice with complete tumor regression per total number of mice in each group. The CYP + AIT group was significantly different from the Nil and CYP groups [F(2,17) = 21.8328, P < 0.0001].

Host Balb/C mice with 4T1 tumors were untreated, treated with CYP on day 9, or treated with CYP followed on day 10 by adoptive transfer of B/I-activated 4T1 DLN cells. As shown in Figure 1, CYP treatment alone briefly slowed tumor progression, but did not lead to complete tumor regression in any mice. Complete tumor regression was seen in 6 out of 6 mice treated with CYP plus adoptive transfer of B/I-activated tumor-sensitized lymphocytes. We have previously shown and published that adoptive transfer of B/I activated 4T1 DLN was ineffective at inducing tumor regression [28,31].

### CD8^+ ^cells are the predominant mediators of tumor regression

**Figure 2:**
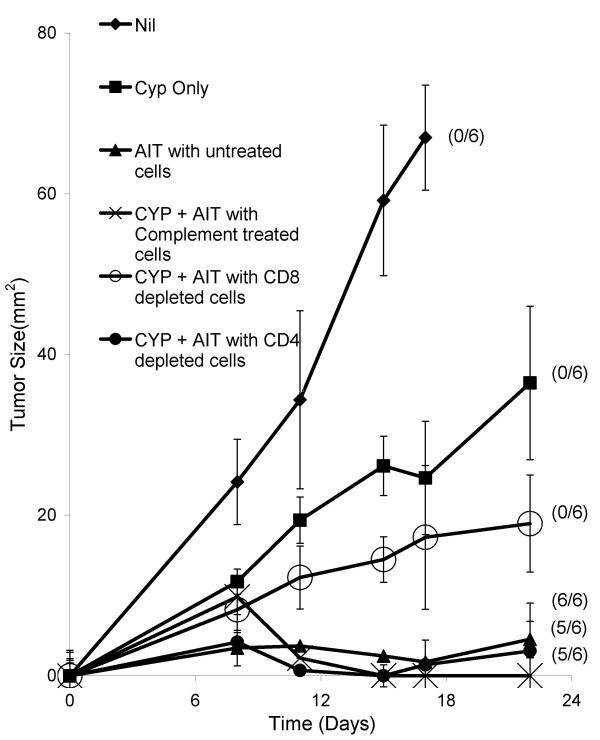
**Therapeutic effect of AIT is mediated predominantly by CD8**^**+ **^**T cells**. Mice with established 4T1 flank tumors were either untreated, treated with CYP alone (on day 3) or CYP on day 3 + AIT on day 4 with 20 × 10^6 ^cells from culture. The cells infused for AIT were either untreated, treated with C' alone, treated with anti-CD8 Ab + C' or with anti-CD4 antibody + C' prior to transfer. Mean tumor sizes ± SE are charted over time. Numbers to the right indicate the number of mice with complete tumor regression per total number of mice in each group. The results with AIT using untreated, C' treated, and CD4-depleted cells were significantly different from the untreated group, the CYP only group, and the CD8-depleted AIT groups [F(4,24) = 32.45, P < 0.0001].

We hypothesized that the predominant cells responsible for tumor regression induced by AIT with B/I-activated tDLN cells would be in the CD8^+ ^subset. By immunohistochemistry, we had previously seen CD4^+ ^and CD8^+ ^T cells infiltrating tumors in mice treated with B/I activated lymphocytes [28]. To determine the relative roles of CD4 and CD8 T cell subsets in inducing tumor regression, BALB/c mice bearing 4 day tumors and pre-treated with CYP, underwent AIT using untreated B/I activated DLN cells, C'-treated cells, anti-CD4 + C'-treated cells, or anti-CD8 + C'-treated cells. In mice treated with CD8-depleted cells, 4T1 tumor growth was depressed slightly compared to CYP alone, but none of the tumors regressed completely, and tumor sizes were not significantly different from CYP alone (Figure 2). In tumor-bearing mice treated with CD4-depleted DLN, 4T1 tumors regressed completely in 5/6 mice, with a growth curve that was little different from AIT with untreated or C'-treated cells.

### Host T cells are not required for tumor regression

To confirm our hypothesis that host T cells play little or no role in tumor regression after adoptive transfer, athymic nude mice with 4 day 4T1 tumors were treated with CYP alone or CYP + B/I activated 4T1 draining lymphocytes, with or without exogenous IL-2 (7500 U i.p. on days 0 - 3 after AIT). Adoptive transfer of B/I activated tDLN was effective at inducing 4T1 tumor regression in nude mice pre-treated with CYP but IL-2 was neither beneficial nor required(data not shown, [F(2,13) = 11.289, P = 0.0014]). This result indicates that host T cells are neither required for tumor regression.

### B/I activated tDLN demonstrate increased expression of message for cytolytic mediators

**Figure 3:**
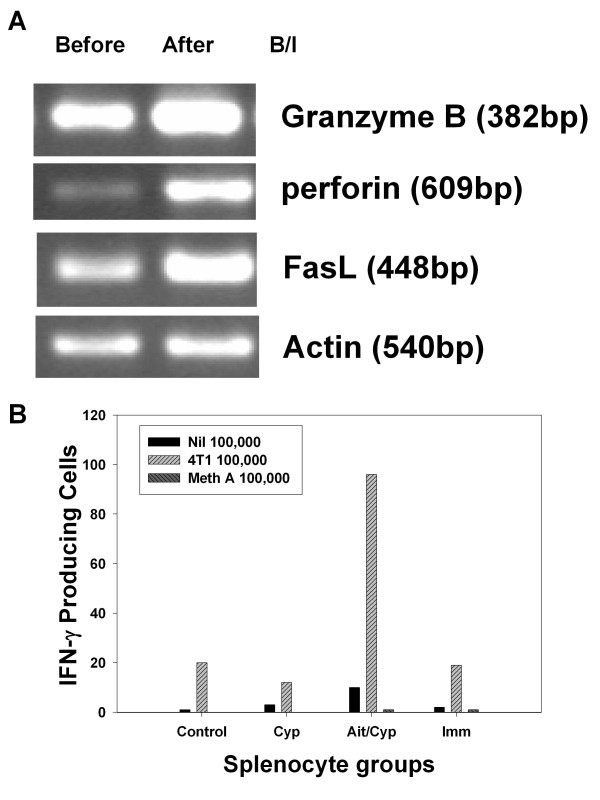
**Adoptive transfer of B/I activated T cells into CYP pretreated hosts results in tumor specific production of interferon-γ and other cytotoxic mediators by adoptive host splenocytes**. A) RT-PCR analysis of granzyme B, perforin, FasL and actin mRNA from murine 4T1 tumor-DLN CD8^+ ^cells, either fresh or after activation with B/I and expansion in IL-2. Increased expression of FasL, perforin, and granzyme B was seen in CD8+ T cells after B/I activation. This is a representative experiment of 3 replicates. B) ELIspot assays for IFN-γ producing cells from spleens of untreated 4T1 tumor-bearing mice, CYP-treated tumor-bearing mice, tumor bearing mice treated with CYP + AIT with B/I-activated tDLN, and 4T1 bearing mice immunized with irradiated 4T1 cells. Splenocytes were unstimulated or stimulated *in vitro *with 4T1 or Meth A sarcoma cells. Results are shown for pooled spleens from 3 mice in each group. Mean values from paired wells are shown. This is a representative experiment of 5 replicates.

We hypothesized that B/I activation and *in vitro *expansion of tumor draining lymphocytes led to the development of highly activated effector T cells, capable of inducing tumor regression. Activation and expansion of tDLN lymphocytes with B/I + IL-2 was associated with increased expression of mRNA for molecules associated with cytotoxic activity, including granzyme B, perforin, and Fas ligand, when compared to unactivated tDLN (Figure 3A).

### B/I activated tDLN release IFN-γ in response to tumor challenge

We and others have previously noted a strong correlation between interferon- IFN-γ production and tumor regression in other tumor models [31-37]. BALB/c mice were either untreated, vaccinated with irradiated 4T1, treated with CYP alone, or treated with CYP + AIT with B/I activated 4T1 tDLN lymphocytes. Spleens were harvested 5 days after adoptive transfer, and pooled splenocytes from 3 mice in each treatment group were co-cultured with irradiated 4T1 or unrelated MethA sarcoma for 24 hours on an IFN-γ ELISpot plate. As shown in Figure 3b, numbers of IFN-**γ **producing cells were greatest in response to 4T1 in mice treated with CYP + AIT. A small number of cells in each of the other groups also produced IFN-**γ **in response to 4T1, which may be explained by sensitization to 4T1 in these tumor-bearing hosts. There was no IFN- **γ **response to Meth A.

### B/I activation leads to increased expression of memory markers by tumor draining lymphocytes

There have recently been reports in the literature that adoptive transfer of central memory or early effector phenotype cells (which are CD62L^high^) is more effective against tumor than adoptive transfer of effector memory or late effector phenotype (CD62L^low ^) T cells [38,39]. Over the course of expansion after B/I activation, we observed upregulation of CD62L by previously CD62L^- ^cells; 70% of the adoptively transferred cells in the B/I-activated cultured tDLN cells were CD62L^+^. In addition, we have previously observed in B/I activated cells minimal levels of cytotoxic activity and high IFN-γ production, which are also consistent with a central memory/early effector phenotype. Therefore, we investigated the hypothesis that activation with B/I could skew the phenotype of activated T cells towards a central memory or early effector phenotype, and that these cells were largely responsible for their anti-tumor efficacy.

In Table 1, expression of phenotypic markers of memory T cells before and after B/I activation is shown for the fraction of tDLN cells that were initially CD62L^-^. 4T1 tDLN were separated into their CD62L subsets by magnetic bead selection. Prior to B/I activation, the CD62L^- ^fraction was 93% CD62L^- ^overall and 81% of the CD8 cells in that fraction were CD62L^-^. After B/I activation and expansion, expression of CD62L, CD127, CD69, and CD27 increased dramatically. These increases in expression of CD127, CD27, CD69, and CD62L are consistent with acquisition of a central memory (T_CM_) phenotype, but we did not observe significant upregulation of CCR7, another memory marker.

**Table 1:** Phenotype of CD62L^-^-enriched T cells from tDLN, before and after B/I pulse and expansion in IL-2

**% of CD8 cells which express**:	Before B/I	After B/I
**CD62L**^**+**^	19%	46%
**CD127**^**+**^	9%	48%
**CD69**^**+**^	45%	90%
**CD27**^**+**^	37%	89%
**CCR7 **^**+**^	46%	42%

4T1 tDLN were harvested and separated by expression of CD62L. Subsets were activated by B/I and expanded for 7 days. Prior to B/I activation, and again after expansion, cells were stained for expression of CD8, CD62L, CD127, CD69, CD27, and CCR7. The CD62L^- ^fraction was initially 93% CD62L^- ^overall. Shown in the table is the percentage of CD8^+ ^cells which express specific T cell markers, before and after B/I expansion, in the initially CD62L^- ^fraction of tDLN cells. Results are representative of more than three independent experiments.

### Before and after B/I activation and expansion, anti-tumor activity resides predominantly in the CD62L^- ^subset

**Figure 4:**
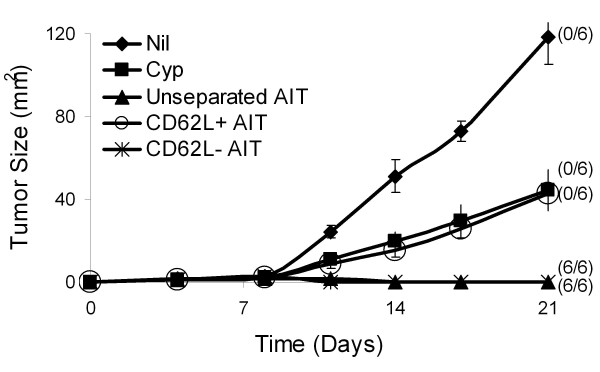
**CD62L phenotype (after B/I activation and expansion) of T cells mediating tumor regression**. Mice with established 4T1 flank tumors were either untreated, treated with CYP alone (100 mg/kg i.p. on day 3) or CYP + AIT (on day 4) with 10 × 10^6 ^unsorted, CD62L^+ ^or CD62^- ^B/I activated tumor-sensitized lymphocytes. CD62L separation was performed after B/I activation and expansion for 7 days in culture, just prior to adoptive transfer. Mean tumor sizes ± SE are charted over time. Numbers to the right indicate the number of mice with complete tumor regression per total number of mice in each group. The AIT and CD62L^- ^AIT groups were significantly different from the untreated group and the CD62L^+ ^AIT groups [F(4,29) = 15.3889, P < 0.0001].

To test the hypothesis that T_CM _cells (which should be CD62L^+^) generated after B/I activation are responsible for the efficacy of these cells at inducing tumor regression, we separated B/I activated and expanded 4T1 tDLN into CD62L^+ ^and CD62L^- ^fractions, using magnetic beads. Unsorted, CD62L^+^, and CD62L^- ^cells were then infused into CYP pre-treated 4T1 tumor bearing mice (Figure 4). Surprisingly, the CD62L^+ ^subset did not induce tumor regression, while the CD62L^- ^subset was highly effective.

**Figure 5:**
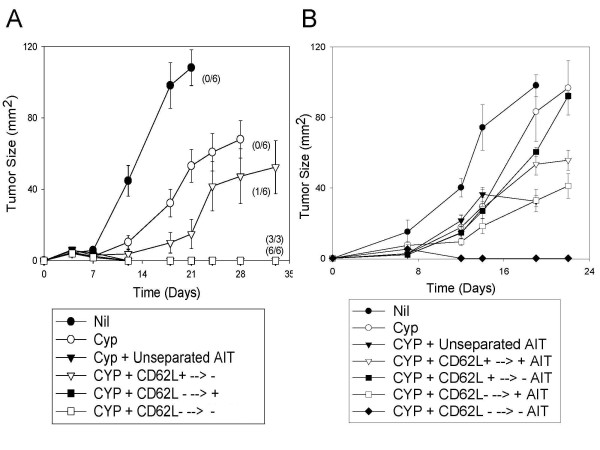
**Anti-tumor activity resides predominantly in the subset of tDLN cells that were initially CD62L**^- ^**and remained CD62L**^- ^**after B/I activation and expansion in culture**. Tumor-sensitized lymphocytes were separated by CD62L phenotype, both before and after B/I activation, resulting in 5 groups: unsorted B/I activated tDLN, CD62L^+ ^cells that remained CD62L^+ ^after B/I activation and expansion (CD62L^+ ^→ CD62L^+ ^), CD62L^+ ^cells that downregulated CD62L after B/I (CD62L^+ ^→ CD62L^- ^), CD62L^- ^cells that remained CD62L^- ^after B/I (CD62L^- ^→CD62L^- ^), and CD62L^- ^cells that upregulated CD62L expression after B/I (CD62L^- ^→ CD62L^+ ^). Mean tumor sizes ± SE are charted over time. Numbers to the right indicate the number of mice with complete tumor regression per total number of mice. A) 4T1 tumor-bearing mice (3 days after inoculation of 50,000 4T1 cells) were either untreated, treated with CYP alone or treated with CYP + AIT (on day 4) with unfractionated or twice fractionated cells, as described above. 20 × 10^6 ^whole, 20 × 10^6 ^(CD62L^+ ^→ CD62L^-^), 4 × 10^6 ^(CD62L^- ^→ CD62L^+^), or 8 × 10^6 ^(CD62L^- ^→ CD62L^- ^) cells were transferred, variations due to the limited number of cells available in subsets. The unseparated, (CD62L^- ^→ CD62L^+^), and (CD62L^- ^→ CD62L^- ^) groups were significantly different from the untreated, CYP only, and (CD62L^+ ^→ CD62L^-^) groups [F(5,32) = 22.9156, P < 0.0001]. B) 4T1 tumor-bearing mice (3 days after inoculation of 100,000 4T1 cells) were either untreated, treated with CYP alone or treated with CYP + AIT (on day 4) with 15 × 10^6 ^unfractionated or twice fractionated cells (except for the (CD62L^- ^→ CD62L^+^) group, which received 10 × 10^6 ^cells). The unseparated and (CD62L^- ^→ CD62L^- ^) groups were significantly different from the untreated, CYP only, (CD62L^- ^→ CD62L^+ ^), and (CD62L^+ ^→ CD62L^- ^) groups [F(6,37) = 17.7496, P < 0.0001].

To characterize not only the ultimate phenotype of anti-tumor effectors after B/I activation, but also the origin of the adoptively transferred cells that mediate the anti-tumor activity, we separated tDLN into CD62L^+ ^and CD62L^- ^fractions, both before and after B/I stimulation and expansion. Unsorted B/I activated lymphocytes, CD62L^+ ^cells that remained CD62L^+ ^after B/I and expansion (CD62L^+ ^→ CD62L^+ ^), CD62L^+ ^cells that downregulated CD62L after B/I treatment and expansion (CD62L^+ ^→ CD62L^- ^), CD62L^- ^cells that remained CD62L^- ^after B/I treatment and expansion (CD62L^- ^→CD62L^- ^), or CD62L^- ^cells that upregulated CD62L expression after B/I and expansion (CD62L^- ^→ CD62L^+ ^) were infused into CYP pre-treated tumor bearing mice (Figure 5). Prior to B/I activation, between 75-83% of lymphocytes are CD62L^+ ^and 17-25% are CD62L^- ^(pooled data from 4 experiments). After B/I activation, from the initially CD62L^+ ^fraction, 87% of the cells remained CD62L^+ ^and 13% downregulated CD62L. From the initially CD62L^- ^fraction, 80% of the cells remained CD62L^- ^and 20% of the cells upregulated CD62L (Representative data from one experiment of 4). In 4 out of 4 experiments, CD62L^- ^→CD62L^- ^cells induced complete tumor regression, even when as few as 375,000 cells per mouse were transferred (Figure 5a, 5b). In 3 out of 4 experiments, CD62L^- ^→CD62L^+ ^cells induced complete tumor regression(Figure 5a). However, when B/I activated lymphocyte subsets were used to treat slightly larger tumors (inoculated at 100,000 cells/mouse, 2x the usual inoculum), CD62L^- ^→CD62L^+ ^cells were not effective at mediating tumor regression, slowing tumor growth only slightly (Figure 5b). In contrast, even with the greater tumor burden, CD62L^- ^→CD62L^- ^cells induced complete tumor regression in all of the treated mice (Figure 5b). In all experiments, CD62L^+ ^→CD62L^- ^cells were incapable of mediating tumor regressions, but with smaller tumor burdens these cells did delay tumor growth modestly (Figure 5a). Finally, adoptive transfer of CD62L^+ ^→ CD62L^+ ^cells was consistently ineffective at inhibiting tumor growth (Figure 5).

### Adoptively transferred cells persist in the tumor-bearing host, accumulating preferentially in the tumor draining lymph nodes

By staining B/I activated DLN lymphocytes with CFSE prior to adoptive transfer, the cells can be "tracked," and proliferation of the adoptively transferred cells can also be measured in the host. At 1 hour, 3, 5, 7, and 12 days after adoptive transfer, we harvested the spleens, lungs, inguinal tDLN, and contralateral lymph node (cLN) from AIT-treated mice. The proportion of CFSE+ cells in the spleen peaked on day 3 at 10.5% of the total splenocytes and then declined rapidly (Table 2). Only a small proportion of CFSE^+ ^cells were found in tumors on day 3, but increased to a maximum of 2% by day 5; CFSE^+ ^cell proportions declined thereafter.

**Table 2:** *In vivo *trafficking and accumulation over time of adoptively transferred cells in tumor-bearing mice

	tDLN	cLN	Tumor	Spleen
**Day 0**	0.7%	0.7%	0.1%	3.0%
**Day 3**	9.2%	5.9%	0.2%	10.5%
**Day 5**	10.1%	7.3%	2.1%	3.1%
**Day 7**	10.3%	6.5%	0.8%	3.4%
**Day 12**	8.4%	8.7%	0.1%	2.1%

Mice with established 4T1 flank tumors were either untreated, treated with CYP alone on day 9, or were treated with CYP on Day 9 followed by AIT with 50 × 10^6 ^CFSE-stained B/I activated 4T1 tDLN (on day 10). At 1 hour, 3, 5, 7, and 12 days after AIT, the tumors, spleens, tDLN, and contralateral LN (cLN) were harvested and analyzed by flow cytometry for CFSE^+ ^cells. This is a representative experiment of 5 replicates. Percentages of CFSE^+ ^cells found in different tissues at various times after AIT ("0" is 1 hour after infusion of cells) are shown

CFSE+ cells in tDLN were 9% of the cells by day 3 and persisted at that level at least until day 12. CFSE^+ ^T cells were seen in the tDLN up to 28 days (2.7%) after AIT (latest date tested, data not shown). Although, CFSE^+ ^cells were 6 to 8% of cells in the contralateral lymph node between days 3 and 12 after AIT, the total number of lymphoid cells in the cDLN was much lower than in the tDLN at all times examined. Thus, the total number of CFSE^+ ^cells in the tDLN was up to 170-fold higher than in the cDLN (Table 3). This suggests that the increased numbers of adoptively transferred cells in the tDLN likely results from selective trafficking and/or increased proliferation.

**Table 3:** Absolute numbers of adoptively transferred cells in lymph nodes of tumor-bearing mice

	Day 3	Day 5	Day 7	Day 10
**tDLN**	4.6 × 10^5^	3.9 × 10^5^	2.8 × 10^5^	2.1 × 10^5^
**cLN**	9.1 × 10^4^	2.3 × 10^**3**^	1.8 × 10^4^	6.4 × 10^4^

Mice with established 4T1 flank tumors were either untreated, treated with CYP alone on day 9, or were treated with CYP on Day 9 after tumor inoculation followed by AIT with 50 × 10^6 ^CFSE-stained B/I activated 4T1 tDLN (on day 10). At 1 hour, 3, 5, 7, and 10 days after AIT, the tDLN and contralateral LN (cLN) were harvested and analyzed by flow cytometry for CFSE^+ ^cells. This is a representative experiment of 5 replicates. Absolute numbers of cells found in each tissue are listed.

Because CFSE dilutes with cell proliferation, we also assessed T cell infiltration in tumors by immunohistochemistry. We observed that tumors from mice treated with AIT using vDLN cells showed infiltration of CD8+ T cells (16-50%) on day 1 after AIT and CD8+ cells persisted at levels under 15% until the last time point checked (day 11). We did not observe infiltration of untreated or CYP treated tumors by CD8+ T cells at any of the time points (data shown in table 4). CD4 infiltrate was seen in all tumor bearing mice, with greater percentages of CD4 infiltrate seen in AIT + CYP treated tumors, as compared to untreated or CYP treated hosts (data not shown).

**Table 4:** CD8+ T cells infiltrate 4T1 tumors after AIT treatment

*Group*	Nil	CD4	CD8a
**Day 1**: Control	-	**+**	-
Cyp	-	**_**	-
AIT/CYP	-	**++**	**++**
**Day 4**: Control	-	**+**	-
CYP	-	**++**	-
AIT/CYP	-	**+++**	**+**
**Day 7**: Control	-	**+**	-
CYP	-	**+**	-
AIT/CYP	-	**+**	**+**
**Day 11**: Control	-	**++**	-
CYP	-	**+**	-
AIT/CYP	-	**+++**	**+**

Grading:

- No stain

+ 1-15%

++ 16-50%

+++ >50%

Mice with established 4T1 flank tumors were either untreated, treated with CYP alone on day 3, or were treated with CYP on Day 3 after tumor inoculation followed by AIT with 25 × 10^6 ^B/I activated 4T1 tDLN (on day 4). At 1, 4, 7 and 11 days after AIT, the tumors were harvested and tumor cross-sections were stained for CD4 and CD8 expression. A representative experiment is shown.

### The trafficking and/or proliferation of adoptively transferred cells in the host is tumor specific

To determine whether the selective accumulation of adoptively transferred cells in the lymphoid organs of adoptive hosts was antigen specific or resulted from non-specific changes caused by growth of a tumor, B/I activated lymphocytes labeled with CFSE were infused into normal mice, 4T1-bearing hosts, and MethA sarcoma bearing-hosts. In the absence of 4T1 tumor antigen, accumulation and/or proliferation of adoptively transferred cells was greatly reduced. On day 3, 10.4% of the splenocytes in 4T1 bearing hosts were CFSE^+^, compared to only 2.1% in MethA bearing hosts and 2.4% in naïve hosts. On day 6, 3.3% of the splenocytes in a 4T1 bearing host were CFSE^+^, but only 0.5% were CFSE^+ ^in a MethA bearing host. Similar results were seen in the tDLN; 9.2% of 4T1 tDLN were CFSE^+ ^on day 3 and 10.1% on day 6. In MethA tDLN, only 3.1% of the cells were CFSE^+ ^on day 3, declining to 2% on day 6 (data summarized in Table 5).

**Table 5:** B/I activated tDLN track preferentially to 4T1 tDLN and persist

Tumor		MethA	4T1	No Tumor
**Day 3**	Spleen	2.1%	10.4%	2.4%
**Day 6**	Spleen	0.5%	3.3%	1.7%
**Day 3**	tDLN	3.1%	9.2%	**_____**
**Day 6**	tDLN	2%	10.1%	**_____**

Naïve mice or mice with established MethA or 4T1 flank tumors were treated with CYP on day 9 followed by AIT with 50 × 10^6 ^CFSE-stained B/I activated 4T1 tDLN (on day 10). At 3 and 6 days after AIT, the tDLN and spleens were harvested and analyzed by flow cytometry for CFSE + cells. This is a representative experiment of 3 replicates.

### Adoptively transferred cells have an activated phenotype in the tumor bearing host (TBH)

**Figure 6:**
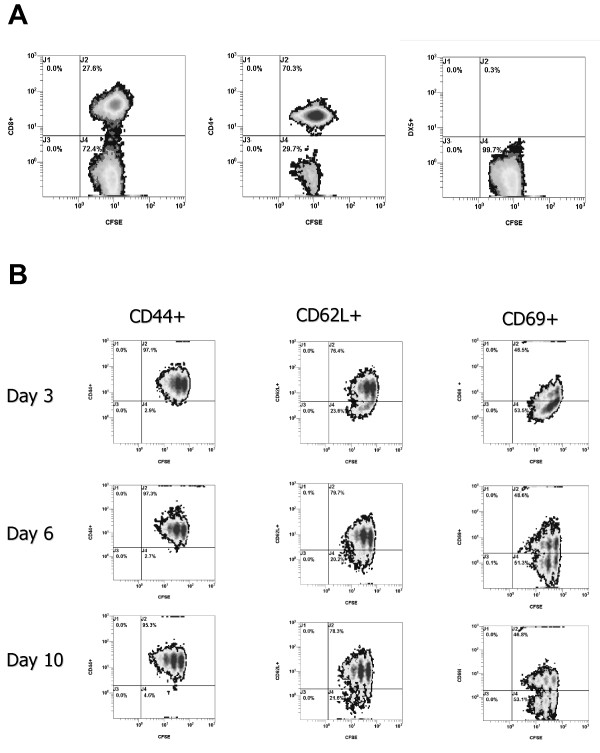
**Phenotype of adoptively transferred cells after infusion into TBH mice **. Mice with established 4T1 flank tumors received CYP (100 mg/kg i.p.) on day 9 after tumor inoculation, followed by AIT with 50 × 10^6 ^B/I activated CFSE-stained lymphocytes on day 10. At days 3, 6 and 10 after AIT, the tDLN were harvested, stained for expression of cell surface markers, and analyzed by flow cytometry. Results shown are gated for CFSE^+ ^cells. A) Dual color analysis of CFSE and CD8 or CD4 or DX5 on Day 3. B) Dual color analysis of CFSE and CD44, CD62L or CD69 on days 3, 6 and 10 after AIT.

In order to determine the phenotypes of the adoptively transferred cells accumulating in the recipient tDLN after AIT with B/I-activated lymphocytes, tDLN were harvested from 4T1 TBH mice at varying times after CYP + AIT with CFSE-labelled B/I-activated lymphocytes. On day 3, 27.6% of CFSE^+ ^cells in the tDLN were CD8^+^, 70.3% were CD4^+^, and 0.3% were DX5^+ ^(Figure 6a). More than 95% of the CFSE^+ ^cells in the tDLN were CD44^+ ^on days 3, 6, and 10. Between 76% and 80% of the CFSE^+ ^cells in the tDLN were CD62L^+^, and 46.5-48.6% of the adoptively transferred cells in the tDLN were CD69^+^. As shown in Figure 6b, three to four distinct generations of CFSE^+ ^cells became evident as time progressed.

### Adoptively transferred cells proliferate in the tumor bearing host, with more generations seen in the tumor draining lymph nodes than in the contrateral nodes

**Figure 7:**
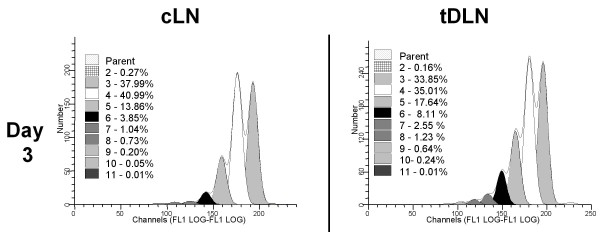
**Adoptively transferred B/I-activated cells proliferate *in vivo***. Mice with established 4T1 flank tumors were treated with CYP on day 9 after tumor inoculation followed by AIT with 50 × 10^6 ^CFSE-stained B/I activated lymphocytes from culture (on day 10). At 3 days after AIT, the tDLN, and contralateral LN (cLN) were harvested and analyzed by flow cytometry for CFSE^+ ^cells. Proliferation and generations of CFSE^+ ^cells were analyzed with ModFit software. Modfit analysis showed the presence of at least 9 cell generations (Reduced chi-squared = 1.238)

With the use of Modfit analysis software, it is possible to determine the number of cycles of proliferation occurring in CFSE labeled cells, and the percentage of cells in each generation. As shown in Figure 7, greater proliferation of the adoptively transferred cells was seen in tDLN, with higher percentages of cells in later generations than in cLN on days 3 and 6 after adoptive transfer. On day 3, 30.42% of the adoptively transferred cells in tDLN were in generations 5 and above, compared to just 19.74% of cells in the cLN. The difference was not as striking on day 6 (67.8 vs. 63.4%, data not shown), but, as shown below, the proliferation is not just dependent on antigen but also may result from the prior stimulation with B/I *in vitro*.

### Proliferation *in vivo *was dependent upon B/I activation before adoptive transfer

**Figure 8:**
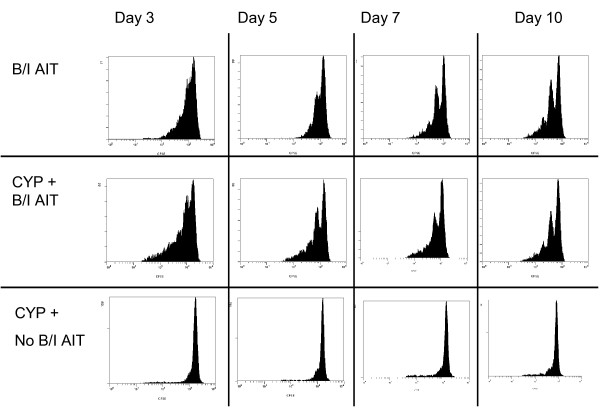
**B/I activation is required for proliferation of adoptively transferred tDLN**. Mice with established 10 day 4T1 flank tumors received 50 × 10^6 ^CFSE-stained lymphocytes, which were either activated with B/I and expanded in culture or untreated ("No B/I"). The mice shown in the upper panel received AIT with cells only; mice in the lower 2 panels received CYP (100 mg/kg i.p.) on day 9 after tumor inoculation. At days 3, 5, 7 and 10 after AIT, the tDLN were harvested from the recipient mice and analyzed by flow cytometry for CFSE^+ ^cells. This is a representative experiment of 3 replicates.

To ascertain whether the proliferation we observed *in vivo *for B/I activated tDLN in TBH mice resulted simply from homeostatic proliferation after the CYP treatment or is enhanced by the prior B/I stimulation, freshly harvested tDLN were transferred into CYP pre-treated TBH mice and B/I activated tDLN were infused into similar hosts, with or without CYP pre-treatment. As shown in Figure 8, adoptively transferred non-activated tDLN did not proliferate significantly, even in mice bearing the relevant tumor and treated with CYP. These cells also did not induce tumor regression (data not shown). In contrast, strong proliferation was seen in B/I activated tDLN transferred into TBH, with or without CYP pre-treatment (Figure 8). Thus, the proliferation of adoptively transferred cells in TBH mice was induced neither by CYP pre-treatment of the host before AIT nor by stimulation with tumor antigen, but required B/I activation.

### How does CYP augment the anti-tumor effect of AIT with B/I-activated lymphocytes?

We observed slightly greater proliferation of adoptively transferred CFSE-labelled cells in the tDLN of CYP pretreated hosts compared to similar hosts not treated with CYP. In CYP pretreated hosts, 9.38% of cells were in generation 6 and 5.26% of cells in generation 7 on day 3, versus 6.63% in generation 6 and 2.5% in generation 7 in mice not treated with CYP. The numbers of adoptively transferred cells in the tDLN of CYP-treated or untreated hosts were roughly similar on days 3 and 7 after adoptive transfer. The number of CFSE^+ ^cells declined by day 10 in the tDLN of un-treated hosts (5 × 10^4 ^cells vs 3 × 10^5 ^cells in CYP pre-treated hosts). CYP pretreatment, therefore, modestly enhanced the proliferation of adoptively transferred cells and the persistence of these cells at later time points in the tDLN.

**Figure 9:**
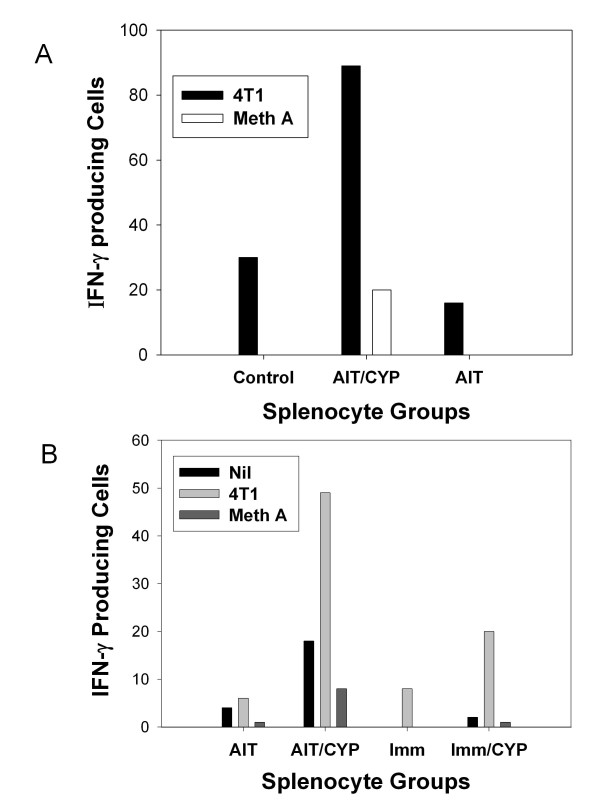
**CYP treatment of AIT recipients increases the number of lymphocytes which produce IFN-γ in response to 4T1 tumor cells**. A) ELIspot assays for IFN-γ producing cells from spleens of mice with established 10 day 4T1 flank tumors which were either untreated, or received AIT with 50 × 10^6 ^B/I activated lymphocytes, with or without CYP pre-treatment (100 mg/kg IP) on day 9. Splenocytes harvested from recipient mice 5 days after AIT were stimulated with 4T1 or MethA sarcoma cells and the number of cells producing IFN-γ were enumerated. Results are pooled from 3 mice in each group, mean values from paired wells are shown, and are representative of 3 replicate experiments B) ELIspot assays for IFN-γ producing cells from spleens of mice with established 10 day 4T1 flank tumors which were either untreated, received AIT with 50 × 10^6 ^B/I activated lymphocytes, with or without CYP pre-treatment (100 mg/kg IP) on day 9, immunized with irradiated 4T1 cells with or without CYP pre-treatment(100 mg/kg IP) on day 9. Splenocytes harvested from recipient mice 8 days after AIT were stimulated with 4T1 or MethA sarcoma cells and the number of cells producing IFN-γ were enumerated. Results are pooled from 3 mice in each group, mean values from paired wells are shown, and are representative of 3 replicate experiments.

In order to determine whether CYP pre-treatment altered the functional response or number of responsive T cells after AIT, the number of cells capable of producing IFN-γ in response to 4T1 tumor in spleens harvested from untreated TBH mice, or TBH mice treated with AIT, with or without CYP pre-treatment, was assessed. As shown in Figure 9A and 9B, the greatest number of cells producing IFN-γ in response to 4T1 tumor was seen in spleens of mice who received CYP prior to adoptive transfer of B/I activated cells.

## Discussion

In this and previous studies, we have demonstrated that B/I activation of tDLN cells produces a population of cells with potent anti-tumor activity against established tumors up to 10 days after inoculation. Even tDLN harvested from donors 20 days post-tumor inoculation, from a metastatic disease state and possibly immunosuppressive milieu, are capable of inducing tumor regression after B/I activation and adoptive transfer into tumor bearing hosts. 14 days after tumor inoculation, 80% of 4T1-tumor bearing mice have measurable metastatic disease, and by 18 days, 100% have lung metastases [31]. We had previously shown that B/I preferentially activated a subset of tDLN cells that were initially CD62L^- ^and that these cells accounted for all of the subsequently developed anti-tumor activity [28]. In the present studies, we have further characterized the post-activation phenotype and the *in vivo *trafficking, and proliferation of the anti-tumor effector cells generated by B/I activation and expansion in culture. The most potent anti-tumor effector cells were highly activated CD8^+ ^T cells which remained CD62L^- ^after B/I activation and expansion. These cells also respond to tumor antigen by specific release of IFN-γ, which we and others have shown correlates with anti-tumor activity [31-37].

Recently, there have been a number of reports that either central memory and/or early effector T cells are the most effective cell types for AIT [38,39]. Conversion of adoptively transferred cells to the central memory phenotype has also been observed in adoptive hosts [40]. T_CM _cells or early effector cells, as opposed to fully differentiated effector or effector memory (T_EM_) cells, have been found to have a greater ability to home to lymphoid tissue via expression of CD62L and CCR7, produce IL-2, proliferate more rapidly in response to antigen and cytokines, and then differentiate into effector cells [41-43]. T_EM _cells lack lymph node homing receptors, exhibit direct (*ex vivo*) cytotoxic activity, do not produce IL-2, and are found in non-lymphoid tissues [44-46]. Thus, T_CM _cells, despite a lower level of immediate effector functions, may be more effective for AIT, because they produce a larger number of anti-tumor cells for a longer period of time. As T cells are repeatedly stimulated, they differentiate from early effector to effector to late effector memory cells. They also downregulate receptors for homeostatic cytokines, and upregulate pro-apoptotic molecules.

We found that after 18 hour B/I activation and 7 days of expansion in IL-2, the initially CD62L^- ^effector cell population harvested from donor mice had largely up-regulated the expression of CD62L (up to 68% CD62L^+ ^). We also observed up-regulation of memory markers CD27, CD44, CD69, and CD127. Furthermore, 76.4% of the adoptively transferred cells that were isolated from the adoptive hosts' tDLN 3 days after AIT were CD62L^+^, which suggested that activation with B/I may stimulate sensitized or CD62L^- ^T_EM _(CD62L^- ^CCR7^low^, CD27^+^, BCL-2 ^hi^) or effector T cells to shift to a CD62L^+ ^T_CM _phenotype, which we initially supposed would be largely responsible for the anti-tumor effects we have observed.

However, we found instead that adoptive transfer of B/I activated and expanded CD62L^- ^cells (separated after expansion in culture) were most effective at mediating tumor regression, and that the CD62L^+ ^fraction had little or no anti-tumor activity. There are a few explanations for these somewhat unexpected results and how it differs from previous reports. For example, this result may reflect enrichment of tumor antigen specific Treg cells in the CD62L^+ ^subset, as recently reported [47]. Alternatively, the CD62L^- ^cells present after B/I activation and expansion may be analogous to secondary response derived memory cells, which are CD62L^- ^[48]. Recent literature suggests a distinct phenotype for T memory cells derived from primary versus those from secondary immune responses [45][48-52]. CD8 T cells undergoing a secondary response expand more rapidly and divide at a faster rate than in a primary response [53]. In the Listeria monocytogenes (LM), Lymphocytic Choriomeningitis Virus (LCMV), and TcR transgenic models, it has been found that secondary immune responses produced memory CD8 T cells which are slow to convert to T_CM_, as measured by both CD62L expression and antigen induced IL-2 production [51]. Primary and secondary response memory CD8 T cells have equal proliferative capacities in respect to numbers of generations, but secondary memory CD8 T cells remain CD62L^low^, in contrast to the CD62L^high ^memory CD8 cells resulting from primary responses. Moreover, secondary CD62L^low ^memory cells are more effective against virulent LM infection and have increased cytolytic activity [48]. The CD62L^- ^T cells harvested from culture after B/I activation and expansion may be more analogous to memory cells derived from a secondary response, which our protocol mimics by re-activation of antigen-sensitized T cells with B/I. This contrasts with the T_EM _or T_CM _cells generated by stimulating naïve pmel-1 TcR transgenic T cells with antigen and cytokines *in vitro *[38,39]. These approaches would generate T_CM _CD8 cells of the primary type, with the secondary immune response not occurring until after adoptive transfer into the tumor bearing host and subsequent *in vivo *vaccination with fowlpox vaccine encoding hgp100 [38,39]. In our model, the primary immune response occurs *in vivo *in the tDLNs of the donor mice, and the secondary immune response occurs *in vitro *upon B/I activation of the tDLN. Our model may be more analagous with the clinical situation, in which sensitized PBMCs, tDLN, or vDLN are isolated from cancer patients, activated and expanded *in vitro*, and secondary memory T cells are then adoptively transferred into patients. Thus, B/I may have the advantage of generating secondary type T memory cells for adoptive transfer.

To further characterize the nature of the anti-tumor effector cells and their CD62L phenotype, tDLN lymphocytes were separated by CD62L phenotype both before and after B/I expansion. The most potent anti-tumor cells were the (CD62L^- ^→CD62L^- ^) cells, which were capable of inducing tumor regression when as few as 375,000 B/I activated cells were transferred and even when double the normal tumor cell inoculum was used to establish the tumors. Studies with CFSE labeled cells showed that the adoptively transferred (CD62L^- ^→CD62L^- ^) subset, despite the lack of CD62L, were able to traffic to tDLN after adoptive transfer, proliferated extensively in the adoptive hosts, and maintained their lack of CD62L expression (data not shown). H The (CD62L^- ^→CD62L^+ ^) subset was less effective than (CD62L^- ^→CD62L^- ^) cells at inducing tumor regression, especially at higher tumor inocula. Surprisingly, (CD62L^+ ^→CD62L^- ^) cells were capable of delaying tumor progression. We previously had not seen any evidence of anti-tumor activity from initially CD62L^+ ^subsets. These (CD62L^+ ^→CD62L^- ^) cells may be tumor specific early effector or memory cells when harvested from donor mice and then may acquire an effector phenotype after exposure to B/I. The existence of these cells may have been masked in previous experiments in which separations were done either before or after B/I activation. When the CD62L separation was carried out only before B/I activation and expansion, they would have been vastly outnumbered by the ineffective (CD62L^+ ^→CD62L^+ ^) subset and possibly suppressed by any Treg (CD4^+ ^CD25^+^) cells also present in that fraction. When CD62L separation was carried out only after B/I activation and expansion, these cells would have been in the CD62L^- ^subset. We have repeated these experiments with similar results, in both the B16 melanoma and B16-OVA models, indicating that this is not a tumor model specific result (data not shown).

Adoptively transferred cells were shown to persist and/or proliferate preferentially in hosts bearing the relevant tumor and accumulated preferentially in tumor draining lymph nodes. The decline in adoptively transferred cells in the spleens after day 3 is consistent with trafficking of these cells to sites of tumor antigen concentration. The low number of CFSE^+ ^cells seen in the tumors may reflect proliferation of the cells beyond the limits of dye detection; after generation 10-12, CFSE becomes too dilute to be readily detected. Using immunohistochemical staining of tumors (not shown), both CD4^+ ^and CD8^+ ^cells were observed to infiltrate into tumors, although CD4^+ ^cells do not appear to be required for tumor regression. Although some of these infiltrating cells could be of host origin, we have found that AIT is effective in nude mice [not shown and [31]], suggesting that host T cells are also not required.

In our model, the increased numbers of adoptively transferred cells in the tDLN suggest that specific uptake and/or proliferation of lymphocytes occurred at the site of antigen and antigen-presenting cells. However, it is not possible to tell how much of the difference in the numbers of cells at the tDLN vs the cLN is due to specific trafficking and accumulation at the site of the tDLN and how much is due to increased proliferation in the tDLN. In fact, we did also observe increased proliferation of the adoptively transferred cells in the tDLN as compared to the cLN, especially on day 3. However, in the absence of specific tumor antigen, adoptively transferred cells did not persist in the host LN. Thus, accumulation of adoptively transferred lymphocytes in the tDLN seems to be specific for tumor antigen, and not merely a result of non-specific inflammation.

In contrast to our trafficking results, others have reported that trafficking and accumulation of infused T cells in TBH mice was indiscriminate, without preference for tumor-draining LN versus other LN [38,39]. It is important to note, however, that after infusion of T cells in those experiments, the adoptive hosts received systemic antigen, in the form of a recombinant fowlpox vaccine, as well as exogenous cytokines. Both of these would be expected to result in stimulation and proliferation of the adoptively transferred cells throughout the adoptive host [38,39]. Furthermore, the TcR transgenic T cells that were infused in that model recognize a self antigen that would be expected to be present throughout the host, not only at the site of tumor growth or the tDLN.

In our model, proliferation of the adoptively transferred cells in the host was also dependent upon prior B/I activation; we did not observe proliferation of unactivated tDLN lymphocytes after adoptive transfer, even in CYP-pretreated hosts. Thus, the proliferation seen in our protocol is a result of B/I activation, and not merely homeostatic proliferation. One of the key potential advantages of B/I activation of tDLN is the ability of these cells to continue proliferating in adoptive hosts after AIT, without the need for exogenous cytokines or antigen vaccination. Interestingly, at the time these cells are harvested from culture, proliferation *in vitro *is generally declining, but apparently accelerates again *in vivo*.

In previous studies, we have shown that adoptively transferred B/I activated lymphocytes were more effective at inducing tumor regression in combination with CYP pre-treatment. The effectiveness of B/I activated lymphocytes with CYP pre-treatment and the lack of requirement for exogenous cytokine therapy is a major advantage of our method of activating tDLN. Several different mechanisms may account for CYP mediated modulation of the immune system. Several studies suggest that CYP causes a breakdown of regulatory mechanisms by removal of a suppressor cell population [54-58]. In early studies, mice that were pre-treated with CYP 2-3 days in advance of sensitization exhibited enhanced contact sensitivity or delayed type hypersensitivity [58-60]. Recent data suggest that CYP may selectively inhibit or deplete CD4+ CD25+ Treg cells [61-65]. CYP may also enhance AIT by temporarily inhibiting tumor growth, by creating "space" for T cell growth, or by a relative increase in cytokines that stimulate T cell proliferation [66].

As noted earlier, in the absence of B/I activation, adoptively transferred cells did not proliferate in CYP pre-treated hosts, indicating that CYP alone does not lead to homeostatic proliferation of adoptively transferred cells and that B/I activation was required for the proliferation of these cells *in vivo*. B/I activated lymphocytes, on the other hand, proliferated in the adoptive hosts with or without CYP pre-treatment. Arguably, slightly more proliferation was seen in CYP pre-treated hosts, but what was more striking was the increase in tumor antigen specific IFN-γ-producing cells in CYP pre-treated hosts compared to untreated hosts. Pre-treatment with CYP before AIT, it would appear, increases the resulting number of IFN-γ producing cells, possibly from the increase in availability of homeostatic cytokines or the removal of suppressive Treg populations.

## Conclusions

B/I activation of tDLN generates large numbers of potent anti-tumor effector tells, as demonstrated by tumor regression *in vivo *and production of IFN-γ *in vitro*. These cells have the ability to traffic to the tumor draining lymph nodes, to proliferate extensively *in vivo *and to mediate tumor regression. Pre-treatment with CYP enhances the efficacy of adoptive immunotherapy, by increasing the number of resulting IFN-γ producing cells. The predominant mediators of anti-tumor activity are CD8^+ ^T cells which are intially CD62L^- ^when harvested from tDLN donors and remain CD62L^- ^after B/I activation and expansion.

The results reported here will be used to distinguish more precisely the most effective anti-tumor populations in the tDLN and to explore methods of generating more of these cells. B/I activation is unique in that it is capable of selectively activating only sensitized T cells, appears to generate highly effective T cells of a secondary memory phenotype, and perhaps for that reason, does not require exogenous cytokines to be administered to the adoptive tumor-bearing host. This could avoid much of the toxicity associated with some AIT regimens. We are currently exploring the use of alternate γ-chain cytokines instead of IL-2 to program even more effectively the phenotypic development of B/I activated tDLN to a T memory phenotype, while decreasing the potential for expanding Treg cells.

## Abbreviations

Abs: antibodies; AIT: adoptive immunotherapy; ANOVA: analysis of variance; B/I: bryostatin and ionomycin; C': complement; CFDA-SE: Carboxyfluorescein Diacetate Succinimidyl Ester; CFSE: carboxyfluoroscein succinimidyl ester; cLN: contralateral lymph node; CTL: cytotoxic T lymphocytes; CYP: cyclophosphamide; DLN: draining lymph node; IFN-γ: Interferon-γ; IP: intraperitoneal; IV: intravenously; LCMV: Lymphocytic Choriomeningitis Virus; LM: Listeria monocytogenes; mAb: monoclonal antibody; PBMC: peripheral blood mononuclear cells; TBH: tumor bearing host; T_CM_: central memory T cells; TcR: T cell receptor; tDLN: tumor draining lymph node; T_EM_: effector memory T cells; Treg: T regulatory cells; Tukey's HSD: Tukey-Kramer honestly significant difference test.

## Authors' contributions

CM carried out the immunotherapy protocols, immunostaining and flow cytometric analysis, carried out the statistical analysis and drafted the manuscript. LG performed the ELIspot assays and participated in the design of the studies. HB conceived of the study, participated in it's design and coordination and helped to draft the manuscript. All authors read and approved of the final manuscript.
